# Cecal Perforation Induced by Migrated Biliary Stent as a Rare Complication of ERCP: A Case Report and Literature Review

**DOI:** 10.1155/2023/9493333

**Published:** 2023-08-11

**Authors:** Arash Mohammadi Tofigh, Hamed Tahmasbi, Majid Iranshahi, Alireza Haghbin Toutounchi, Hojatolah Khoshnoudi, Seyed Pedram Kouchak Hosseini

**Affiliations:** ^1^Department of General Surgery, Imam Hosein Medical and Educational Center, Shahid Beheshti University of Medical Sciences, Tehran, Iran; ^2^Gastroenterology and Liver Diseases Research Center, Shahid Beheshti University of Medical Sciences, Tehran, Iran

## Abstract

*Introduction and Importance.* Endoscopic retrograde cholangiopancreatography (ERCP) is a non-surgical method utilized to manage biliary tract obstruction, but the complication of biliary stent migration occurs in 5–10% of patients. Though migrated stents are commonly passed through the gastrointestinal tract without harm, intestinal perforation is a rare but severe complication, affecting less than 1% of cases. *Case Presentation.* We report a case of a 65-year-old woman who presented to the emergency department with symptoms of abdominal pain, nausea, and loss of appetite. According to clinical examination and evidence, the patient underwent surgery with high suspicion of appendicitis, which unexpectedly uncovered a perforated cecum with a protruding biliary stent. *Clinical Discussion.* Our report describes a unique and unexpected finding of cecal perforation caused by a migrated biliary stent in a patient. We also conducted a review of current literature on ERCP complications, including risk factors for stent migration, relevant statistics, and appropriate interventions. *Conclusion.* Surgeons should be aware of the risk of stent migration and complications in patients with a history of ERCP. Removal of migrated biliary stents is recommended, regardless of the presence of complications. Additional assessments for alternative diagnoses are recommended for older patients with abdominal pain complaints. Flexible plastic stents should be used for patients at risk of stent passage.

## 1. Introduction

Endoscopic retrograde cholangiopancreatography (ERCP) is a commonly used non-surgical procedure for the management of biliary tract obstruction [1]. Although effective, one of the known complications of ERCP is the migration of biliary stents, which occurs in 5–10% of patients [2]. While the migrated stent typically passes through the gastrointestinal tract without any complications, intestinal perforation is a rare but severe complication, occurring in less than 1% of cases [2]. In this case, we present a patient with a history of ERCP stenting who initially presented with symptoms of appendicitis, but further assessment revealed a cecal perforation resulting from a migrated biliary stent. Furthermore, we review recent literature regarding ERCP complications, potential risk factors for biliary stent migration, relevant statistical data, and possible interventions. This study has been reported in line with the SCARE 2020 criteria [3].

## 2. Presentation of Case

**Figure 1:**
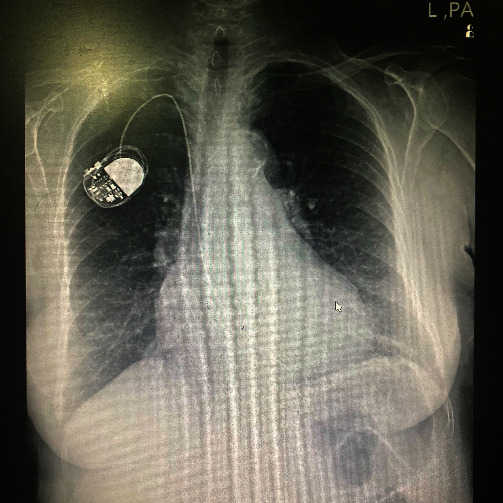
Upright chest X-ray without subdiaphragmatic free air.

**Figure 2:**
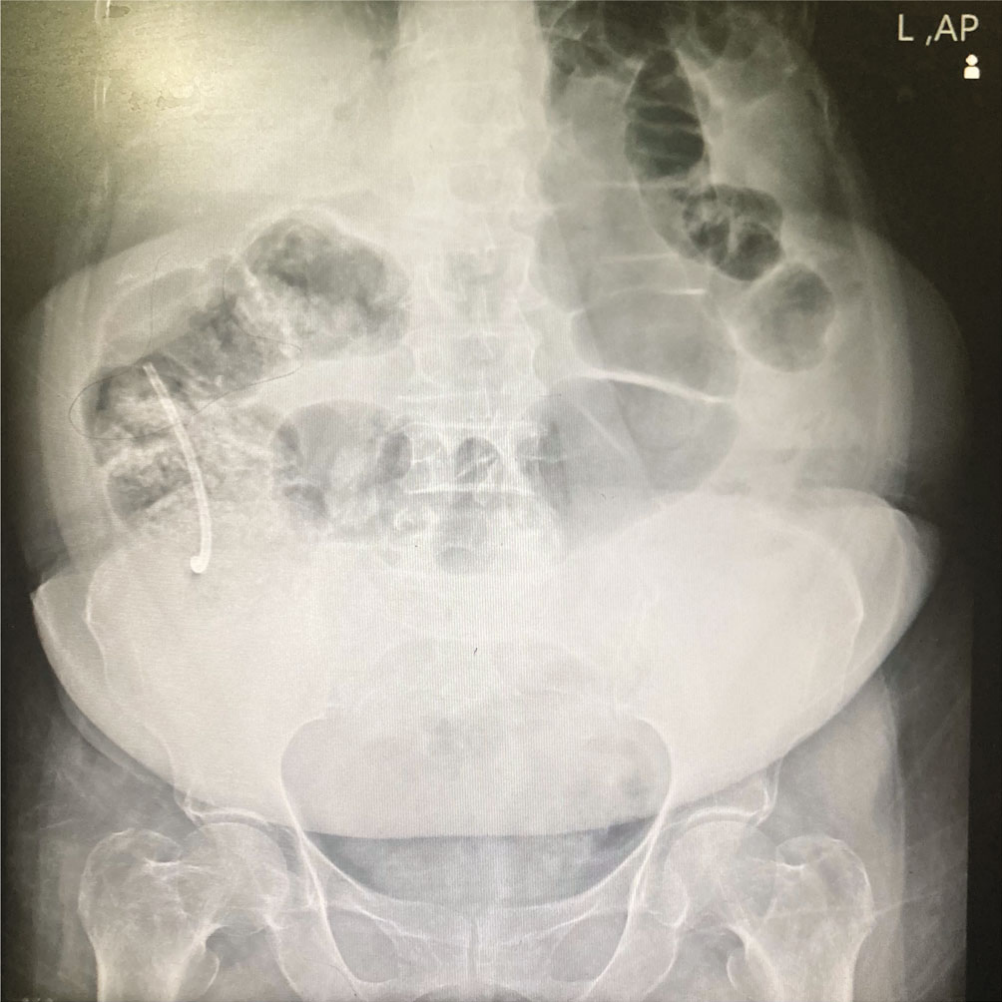
Abdominal radiography showed the stent at the RLQ.

A 65-year-old female patient presented to the emergency department complaining of abdominal pain that had started two days earlier. The patient reported diffuse pain in the right lower abdomen without any shifting of pain, which worsened over the course of a day. The patient also experienced nausea and loss of appetite, but no vomiting, and had no history or evidence of obstruction or obstipation. The patient's vital signs were Blood Pressure: 120/85, Pulse Rate: 90, and oxygen saturation: 99%. The patient's medical history, included a biliary stent placed through ERCP to manage a common bile duct (CBD) stone over three years ago, followed by laparoscopic cholecystectomy. The patient had controlled hypertension and a pacemaker but no history of smoking, familial chronic diseases, cancer, or psychological issues, and had a good educational background. Upon examination, the patient was febrile with a temperature of 38°C and showed tenderness and rebound tenderness on the right lower abdomen. However, no distention, guarding, or palpable mass was detected. Laboratory results indicated a white blood cell count of 13 with 87% thousand/mm^3^ neutrophils and an elevated C-reactive protein (CRP) level of up to 50 mg/dL. A positive Rovsing's sign was also noted. The modified Alvarado scale was calculated, resulting in a score of 8 out of 9. Ultrasound sonography revealed fat stranding at the right lower quadrant with mild free fluid, and no non-compressible loop was detected. A chest X-ray did not show any free intraperitoneal air below the diaphragm. However, an unexpected opaque object was visible in the right lower abdomen on the abdominal radiography, which could be a migrated biliary stent due to the patient's history of ERCP, even if the patient did not mention previous stent placement and denied ingesting any foreign body (Figures 1 and 2). An abdominal computerized tomography (CT) scan was planned, but unfortunately, it was unavailable at that time. Based on the findings, potential diagnoses, such as appendicitis, right colon diverticulitis, or perforation, were considered, respectively. However, based on the positive appendicitis findings and high suspicion of appendicitis, the patient underwent surgery.

**Figure 3:**
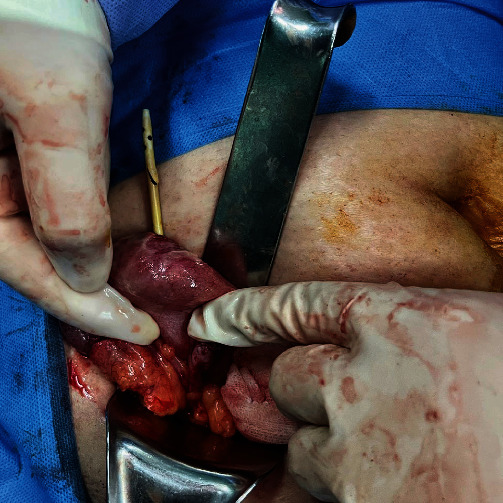
The perforation site at the cecum in exploration through the McBurney incision.

**Figure 4:**
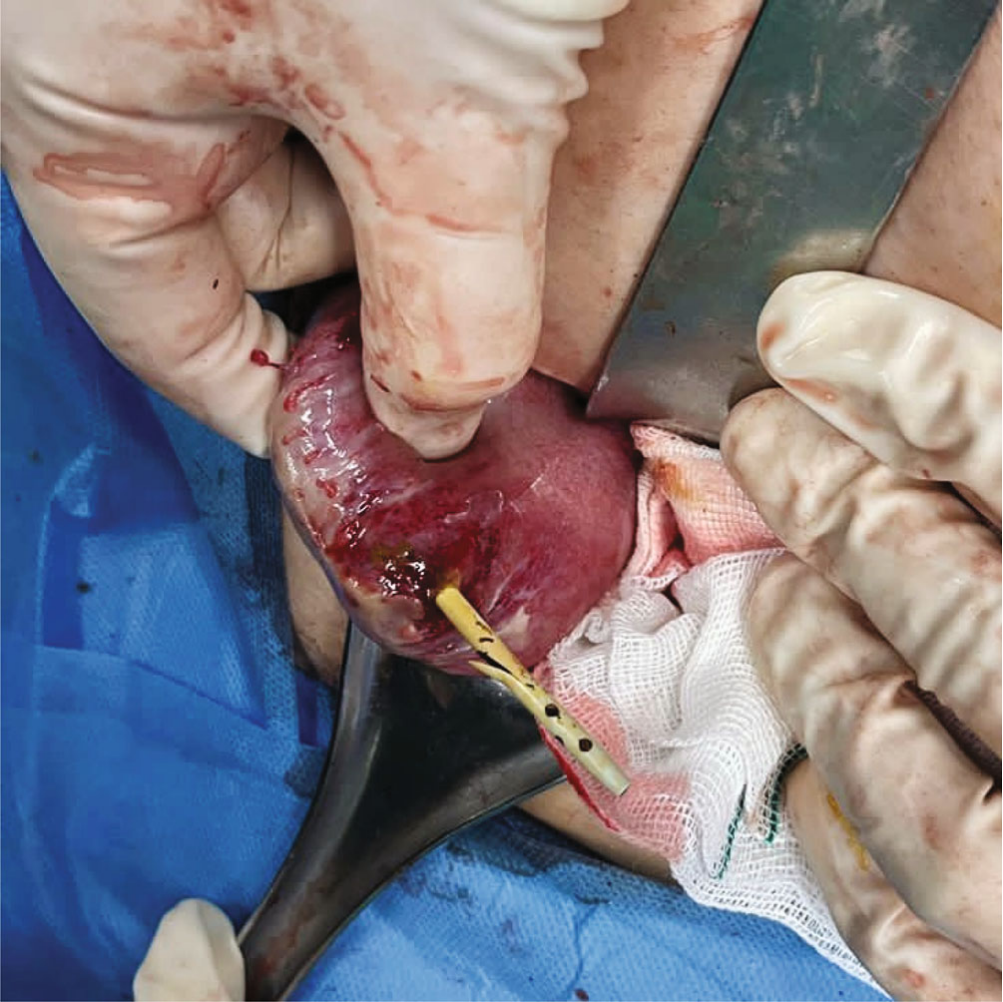
Protruding biliary stent (a rigid plastic straight-type).

**Figure 5:**
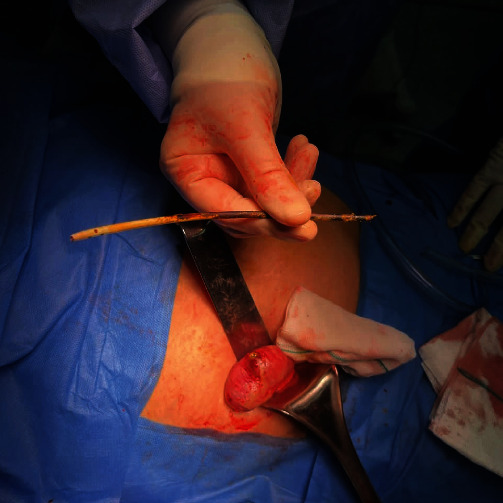
Pulled out ERCP stent from the cecum.

A surgical team consisting of an attending general surgeon and a senior resident operated under general anesthesia via a McBurney incision. Upon exploration, an unexpected perforation was discovered on the cecum's anti-mesenteric site, 1 cm above the ileocecal valve, with a protruding biliary stent (a rigid plastic straight-type; Figures 3 and 4). The perforation site was found to be free of fecal contamination, and the appendix was not inflamed. The incision was extended by 2 cm upwards, and the cecum was mobilized by releasing the white line. The stent was removed (Figure 5), and the mesocolon was ligated. The lumen was resected from 5 cm proximal to the ileocecal valve to 2 cm above the cecum's perforation site, and an ileocolic side-to-side anastomosis was performed using a stapler. The stapled line was then reinforced with PDS 3-0 sutures.

Post-operatively, the patient's condition improved, with leukocytosis decreasing and symptoms being relieved. Her bowel function returned to normal on the fifth day, and she was able to tolerate a regular diet with no nausea or vomiting. There were no signs of leakage or failure of the anastomoses, and she was discharged in good general condition.

## 3. Discussion

Biliary obstruction is a serious condition caused by malignant or benign factors, such as chronic pancreatitis, lithiasis, postoperative strictures, hepatobiliary malignancies, or tension of tumors on the biliary tract. [4] Various approaches can be used to alleviate biliary obstruction based on the patient's condition and indications. Although cholecystojejunostomy and choledochojejunostomy are the primary surgical procedures, they are associated with several complications and require a significant surgical operation on the patient. On the other hand, ERCP is a less invasive solution and the most common non-surgical procedure for biliary tract obstruction [1]. Biliary obstruction may be relieved through the placement of a biliary stent [4].

However, biliary stenting can lead to several complications, including cholecystitis or cholangitis caused by stent occlusion, pancreatitis from duct manipulation, hemorrhage, stent fracture, and stent migration [5]. Biliary stent migration is a common occurrence with a rate of 5–10%, which happens in 3% of patients in the early stage and up to 17% later [2, 6]. Kawaguchi et al. stated that the potential risk factors for stent migration include stents with large diameters, long biliary stents, straight-type stents, stent duration >1 month, CBD diameter >10 mm, and history of sphincterotomy [7]. A retrospective cohort study showed that biliary stent migration occurred more frequently in benign types of biliary obstruction [8].

Biliary stent migration can be classified into proximal and distal types [6]. Distally migrated stents are usually passed spontaneously through the gastrointestinal tract or can be retrieved using endoscopy and fluoroscopy, but less than 1% may cause intestinal perforation, which is rare [2, 4]. The risk of bowel perforation in stent migration is associated with structural bowel abnormalities or variations, such as postoperative bowel adhesion, diverticula, abdominal wall hernias, or strictures [8]. According to Zorbas et al., a systematic review of literature from 2000 until 2020 found 81 cases of bowel perforation induced by migrated biliary stents. The majority of patients had a plastic stent (93.6%). From the total population, 35 patients (44.9%) had duodenal perforation, 23 patients (29.5%) had large bowel perforation, and 18 patients (23.1%) had small bowel perforation [6]. They showed that the most common types of stents causing bowel perforation are plastic stents, and the most common perforation site is the duodenum. The mortality rate of bowel perforation from stent migration was reported to be as high as 10.3%. In addition, Park et al. have analyzed 30 cases of colon perforation caused by distal migrated biliary stents. They reported that most cases were associated with colonic diverticulum (20 out of 30 cases), and the most commonly involved colonic segment was the sigmoid colon (25 sigmoid colons, 1 cecum, 1 ascending colon, 1 splenic flexure, 1 rectum, and 1 appendix) [8].

The primary treatment for migrated biliary stents is surgical removal. However, there are several alternative interventions available, such as percutaneous extraction, endoscopic removal, and mucosal repair, which can also be considered in certain cases [6]. Surgical repair can be performed using open or laparoscopic approaches, depending on the individual patient's clinical status. It is crucial to remove any migrated biliary stent immediately, regardless of the patient's clinical condition, as recommended in the literature [6]. Medical centers should schedule a follow-up appointment for stent removal when the patient's biliary obstruction symptoms are relieved. Furthermore, the literature suggests that the removal of a migrated biliary stent is necessary due to the risk of perforation [6]. Hence, healthcare professionals should prioritize prompt and appropriate management of migrated biliary stents to avoid complications and ensure favorable patient outcomes. In this case, ERCP and stenting were not performed in our center, and we had no access to documents. However, it was a forgotten biliary stent over three years, and he was not followed up for removal. Now retrospectively, a laparoscopic plane seems to be a better choice for this case, but we performed a McBurney incision for possible appendectomy according to high suspicion of appendicitis due to the above evidence and we were just guessing about a biliary stent and relevant complications. Significantly, performing a CT scan could change the diagnosis, probabilities, and surgical plans, but it was not accessible at that time, therefore, we just have to rely on our clinical findings.

Acute appendicitis is typically characterized by symptoms, such as nausea, vomiting, anorexia, and leukocytosis with an elevated neutrophil ratio, in laboratory results. Additionally, localized tenderness, muscular rigidity, and rebound tenderness in the right lower abdomen are also common symptoms [9]. The mean age of individuals with acute appendicitis is 25 years, with 6% of cases occurring in those under the age of 10 years, and 1.3% occurring in individuals over the age of 60 years [5]. However, it is important to note that further evaluation is recommended in elderly patients who present with complaints of abdominal pain, even if it is a typical presentation of appendicitis.

In patients with a history of ERCP, there is a risk of stent migration and associated complications. Therefore, surgeons should consider this possibility in these patients and take appropriate precautions. It is essential to carefully assess the patient's history and medical records to determine the risk of stent migration and other potential complications in patients with a history of ERCP. In patients with a history of ERCP, stent migration is a potential complication that must be considered, and appropriate measures taken to prevent adverse outcomes.

The clinical manifestations of bowel perforation usually involve widespread tenderness, tachycardia, tachypnea, fever, and other indications of peritonitis. Additionally, patients may exhibit nonspecific findings, including leukocytosis, elevated amylase levels, or elevated CRP levels, which lack diagnostic significance. Furthermore, the presence of free intraperitoneal air below the diaphragm on an upright chest X-ray suggests bowel perforation [10]. Overall, in this particular case, a simple abdominal radiograph showed an unusual opaque body, which could be a migrated biliary stent due to the patient's history of ERCP, but we had no access to his medical documents as it was not performed in this center, and he did not mention the establishment of a biliary stent too.

## 4. Conclusion

Surgeons must be aware of the potential for stent migration and complications in patients with a history of ERCP. In cases where a migrated biliary stent is discovered, it should be removed, regardless of the presence or absence of associated complications. For older patients with abdominal pain complaints, additional assessments for other possible diagnoses are recommended. Furthermore, the use of soft and flexible plastic stents is advisable for patients who are at risk of stent passage.

## Data Availability

Data supporting this research article are available from the corresponding author or the first author on reasonable request.
